# Development of an experimental method of systematically estimating protein expression limits in HEK293 cells

**DOI:** 10.1038/s41598-020-61646-3

**Published:** 2020-03-16

**Authors:** Yoshihiro Mori, Yuki Yoshida, Ayano Satoh, Hisao Moriya

**Affiliations:** 1 Graduate School of Natural Science and Technology, Okayama University, Okayama, Japan; 2 Sony Computer Science Laboratories, Tokyo, Japan; 3 Graduate School of Interdisciplinary Science and Engineering in Health Systems, Okayama University, Okayama, Japan; 4 Research Core for Interdisciplinary Sciences, Okayama University, Okayama, Japan; 5 Graduate School of Environmental and Life Science, Okayama University, Okayama, Japan

**Keywords:** Gene expression analysis, Biological techniques, Molecular biology, Cell biology, Protein transport, Protein translocation

## Abstract

Protein overexpression sometimes causes cellular defects, although the underlying mechanism is still unknown. A protein’s expression limit, which triggers cellular defects, is a useful indication of the underlying mechanism. In this study, we developed an experimental method of estimating the expression limits of target proteins in the human embryonic kidney cell line HEK293 by measuring the proteins’ expression levels in cells that survived after the high-copy introduction of plasmid DNA by which the proteins were expressed under a strong cytomegalovirus promoter. The expression limits of nonfluorescent target proteins were indirectly estimated by measuring the levels of green fluorescent protein (GFP) connected to the target proteins with the self-cleaving sequence P2A. The expression limit of a model GFP was ~5.0% of the total protein, and sustained GFP overexpression caused cell death. The expression limits of GFPs with mitochondria-targeting signals and endoplasmic reticulum localization signals were 1.6% and 0.38%, respectively. The expression limits of four proteins involved in vesicular trafficking were far lower compared to a red fluorescent protein. The protein expression limit estimation method developed will be valuable for defining toxic proteins and consequences of protein overexpression.

## Introduction

Protein overexpression sometimes causes cellular defects^1,2^. Although the underlying mechanism causing these defects is still unknown, it can be estimated using the protein expression limit, which triggers cellular defects^3,4^. For example, a protein with the highest expression limit is considered harmless, and its ultimate overexpression causes overloading of protein synthesis^5,6^. Transported proteins should have lower expression limits than cytoplasmic proteins because the overexpression of transported proteins overloads resources for protein transport^5,7^.

We previously developed an experimental genetic tug-of-war (gTOW) method of estimating the expression limits of target proteins in yeasts^8–10^. The expression limits obtained were useful for classifying the mechanisms underlying cellular defects triggered by protein overexpression^4,11^. In this study, we developed an experimental method of systematically evaluating the expression limits of target proteins in the human embryonic kidney cell line HEK293 by measuring the target protein level in cells surviving after high-efficient, multicopy introduction of plasmid DNA by which the target protein is highly expressed under a cytomegalovirus promoter (CMV-pro).

For proof-of-concept, we estimated the expression limits of a green fluorescent protein (GFP) and its derivatives as model proteins. We also indirectly estimated the expression limits of nonfluorescent target proteins by measuring the levels of GFP connected to the target proteins with the self-cleaving sequence P2A by measuring GFP fluorescence. Estimation of expression limits slightly but significantly improved by concentrating cells with higher expression levels of the target protein using the antagonism between dihydrofolate reductase (DHFR) and its inhibitor methotrexate (MTX).

## Results

### Estimation of GFP expression limit with high-efficient transfection

To develop an experimental method of measuring a target protein’s expression limit, we used a GFP as a target protein because its expression level is easily estimated by fluorescence of the cells expressing it. We used moxGFP, which folds quickly and does not misfold in the endoplasmic reticulum (ER)^12^. GFP is considered a nonharmful protein^13–15^, and its expression limit in yeast is ~15% of the total protein, which is highest among other proteins in yeast^5,7^. Therefore, if an experimental condition is established that can estimate the expression limit of GFP, it should be applicable to the majority of other proteins with lower expression limits compared to GFP.

**Figure 1:**
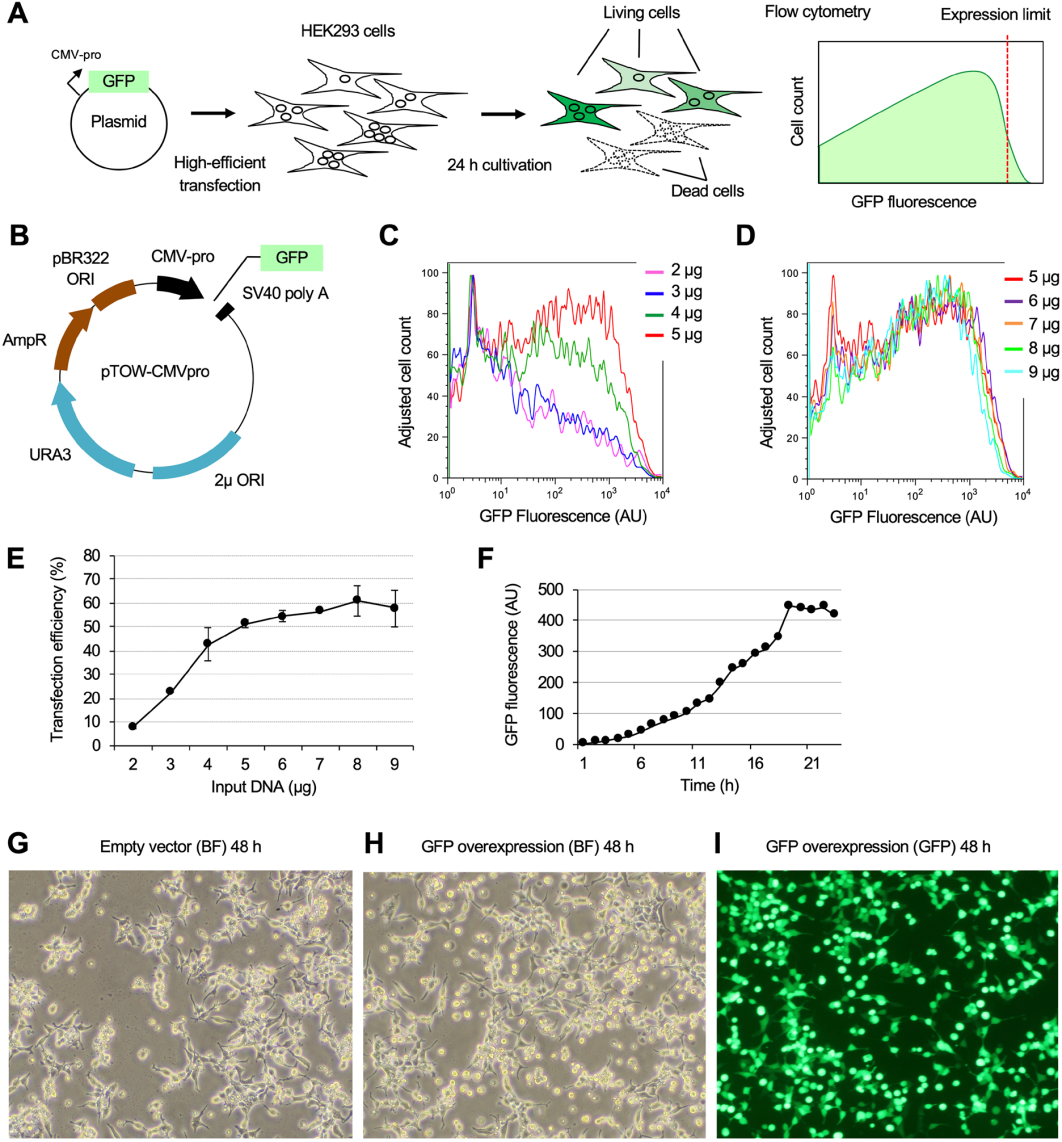
Experimental conditions to estimate GFP expression limit in HEK293 cells. (**A**) Experimental procedure. HEK293 cells are transfected with a plasmid for GFP expression with a high-efficient transfection. After 24 h of transfection, surviving cells are analyzed by flow cytometry. If the promoter strength and transfection efficiency are high enough, the GFP expression levels in the transfected cells will reach the expression limit, which is observed as the maximum boundary of GFP fluorescence in flow cytometry (shown as a virtual histogram). (**B**) Basic structure of plasmids used in this study. For transient transfection, the plasmid does not have any replication origin in cultured cells. The plasmid contains *URA3* and *2µORI* for recombination-based plasmid construction in the budding yeast *Saccharomyces cerevisiae* beside *AmpR* and *pBR322ORI* for selection and amplification in *Escherichia coli*. These factors do not have any specific function in cultured cells. The target gene (here GFP) is expressed under the control of CMV-pro. (**C**,**D**) Effect of DNA amount on GFP expression level in transfected cells. The GFP intensity (AU) of the cell population after 24 h of transfection was analyzed using flow cytometry. DNA amounts used in the transfection are shown. (**E**) Effect of DNA amount on transfection efficiency. The transfection efficiency was calculated as the ratio of GFP-positive cells to the total cell count (20,000) from the histogram shown in (**C**,**D**). The means and SD (error bar) are shown. (**F**) Time course of GFP expression in transfected cells. The cells were analyzed using flow cytometry every hour after transfection with 6 µg of DNA. The GFP fluorescence intensity means of the cell population are shown. (**G**–**I**) Microscopic image of cells transfected with the empty vector (pTOW-CMV-pro, **G**) and the GFP expression plasmid (pTOW-CMV-pro-GFP, **H**,**I**). BF and GFP indicate the phase contrast bright field and GFP fluorescence, respectively. Cells were observed using a 20-fold objective lens. The flow cytometry histograms were created using the FlowJo software (https://www.flowjo.com/) ver. 8.8.7.

Figure 1A shows the experimental procedure. The basic principle of this analysis is that we can assume that transfection creates a cell population harboring diverse plasmid copy numbers, leading to diverse expression levels of a target protein encoded on the plasmid, because the process is random. If the transfection efficiency is high enough, the transfected cell population should contain cells close to or exceeding the target protein’s expression limit. As the growth of cells exceeding the target protein’s expression limit is inhibited, those cells should be eliminated from the population. Therefore, the target protein’s expression limit should be observed as the highest boundary in the fluorescence of the cell population surviving after a certain period of transfection. We used HEK293 cells^16,17^ because their transfection efficiency using a transfection reagent polyethyleneimine (PEI) “Max” is high enough for our purpose^18^. We used CMV-pro because it is widely recognized as a strong promoter in cultured cell lines^19,20^. The basic structure of the plasmid pTOW-CMV-pro we used in this study is shown in Fig. 1B.

We optimized the amount of plasmid DNA for transfection and the analysis timing post-transfection. The percentage of cells with higher GFP fluorescence increased with increasing amounts of DNA used for transfection (Fig. 1C). The percentage and highest boundary did not change with >5 µg DNA (Fig. 1D). The transfection efficiency (ratio of GFP-positive cells to the total cell count of 20,000) also increased with increasing amounts of DNA but was saturated after 6 µg DNA (Fig. 1E). Therefore, with >5 µg DNA, a sufficient amount of plasmid DNA was introduced into cells to provide the expression limit of GFP. The GFP fluorescence of cells reached the maximum value after 18 h of transfection and was maintained thereafter (Fig. 1F). Therefore, we fixed the experimental conditions as follows: 6 µg of DNA used for transfection and analysis of cells 24 h post-transfection.

Cells expressing a high level of GFP became spherical shaped and detached from the bottom of the culture dish (Fig. 1H,I), and these cells died after 72 h (data not shown). These cells were hardly observed in the empty vector transfection (Fig. 1G). Therefore, GFP overexpression (i.e., more than its expression limit) causes cell death.

### Localization to mitochondria and the ER decreases the GFP expression limit

**Figure 2:**
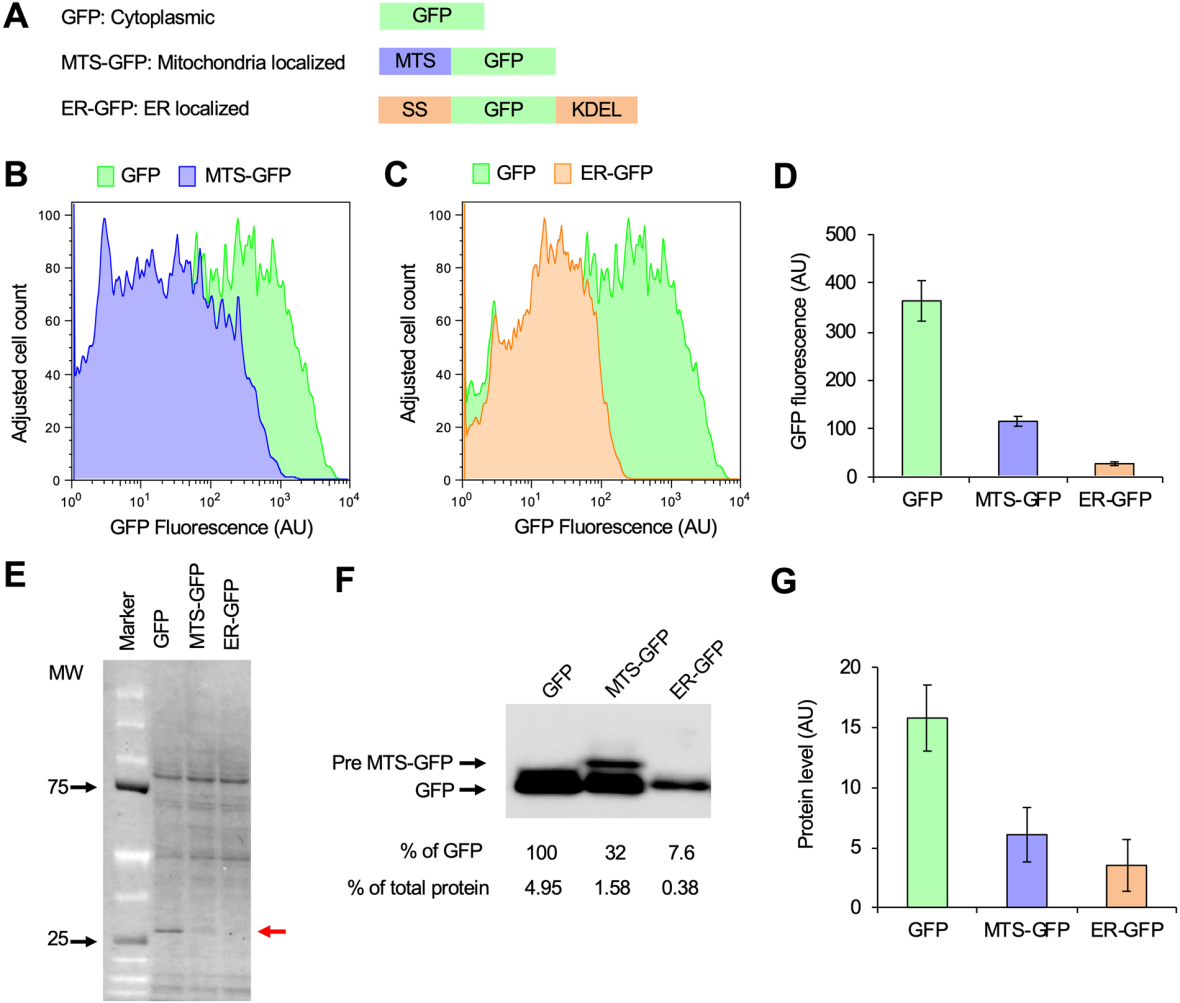
Attachment of localization signals reduces the GFP expression limit. (**A**) Insert constructs used in the experiment. The MTS from human mitochondrial protein Cox8, the SS sequence from *Trypanosoma brucei* EP protein, and the ER retention signal KDEL were attached to GFP, as shown. (**B**–**D**) GFP expression levels of transfected HEK293 cells analyzed using flow cytometry. Histograms of GFP fluorescence (**B**,**C**), and the means and SD (error bar) are shown (**D**). (**E**) GFP expression levels of transfected cells analyzed using protein analysis. The total protein of each transfected cell was separated by SDS-PAGE. The red arrow indicates the size corresponding to GFP. (**F**,**G**) GFP expressed in transfected cells detected using western blotting with an anti-GFP antibody. The band corresponding to the MTS-GFP precursor size is shown as Pre MTS-GFP. Measured intensities of the bands are shown as % of the band of the GFP experiment. The means and SDs from triplicated experiments are calculated and shown in (**G**). The uncropped image of (**F**) is attached as Fig. S3B. The flow cytometry histograms were created using the FlowJo software (https://www.flowjo.com/) ver. 8.8.7.

Previous studies have reported that adding localization signals to GFP decreases its expression limit in yeast, probably because of overloading of localization^7^. In this study, we investigated whether localization also decreases the expression limit of GFP in HEK293 cells. We attached a mitochondrial targeting signal at the N-terminal construct mitochondrial targeting sequence–GFP (MTS-GFP), an ER localization signal at the N-terminal of GFP, and the ER retention signal KDEL at the C-terminal of GFP to construct ER-GFP (Fig. 2A).

Cells transfected with the plasmids harboring MTS-GFP or ER-GFP showed lower maximum GFP fluorescence compared to cells transfected by plasmids harboring GFP (Fig. 2B,C). The fluorescence intensity means were higher in the order of GFP > MTS-GFP > ER-GFP (Fig. 2D). The GFP expressed was observed as a visible band when fluorescently labeled whole cellular proteins were separated by sodium dodecyl sulfate–polyacrylamide gel electrophoresis (SDS-PAGE) (Fig. 2E, red arrow). The GFP expression level estimated from the band intensity was ~4.95% (standard deviation [SD] = 0.78) of the total protein, while MTS-GFP and ER-GFP expression levels estimated from the band intensity of western blotting with an anti-GFP antibody were 32% and 7.6% of the GFP expression level and therefore 1.58% and 0.38% of the total protein, respectively (Fig. 2F,G). In the western blotting of MTS-GFP, we observed a band corresponding to the MTS-GFP precursor (Fig. 2F), indicating that the MTS-GFP expression level in this study actually exceeded the limit of the mitochondrial transport process. GFP localization to mitochondria and the ER therefore decreased expression limits in HEK293 cells, as observed in yeast.

### Estimation of expression limits of nonfluorescent proteins using P2A-GFP

**Figure 3:**
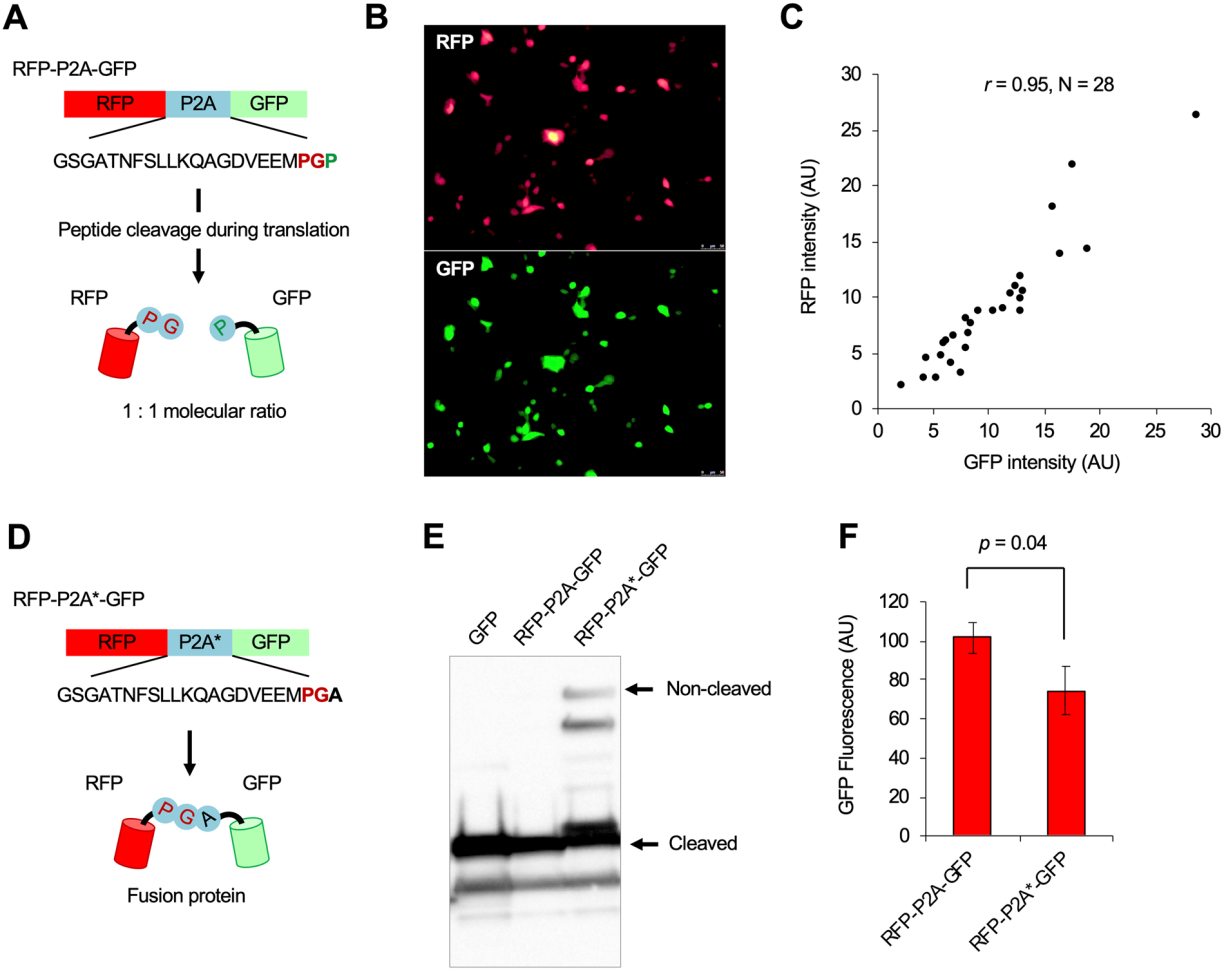
Use of P2A-GFP for estimating the expression levels of N-terminal target proteins. (**A**) Concept of the experiment. Equimolar protein expression can be performed using the P2A sequence. Expression level of an N-terminal target protein (here RFP) thus can be indirectly estimated by the GFP expression level connected at the C-terminal after P2A peptide. (**B**) Microscopic analysis of transfected HEK293 cells. RFP and GFP fluorescence images were taken using a fluorescent microscope, and fluorescence intensities of cells were measured. We confirmed that each of RFP and GFP fluorescence was not leaked to the other fluorescent image (data not shown). (**C**) A scatter plot between RFP and GFP intensities. The Pearson correlation coefficient (*r* = 0.95) is shown. (**D**) Effect of noncleaved mutation in P2A peptide. A mutation in the P2A mutant (RFP-P2A*-GFP) resulted in the formation of a fusion protein. (**E**) Detection of GFP in the cell extract of GFP, RFP-P2A-GFP, and RFP-P2A*-GFP expressing cells using western blotting with an anti-GFP antibody. The corresponding bands of GFP and noncleaved RFP-P2A-GFP are shown by arrowheads and indicated as “Cleaved” and “Noncleaved,” respectively. (**F**) Comparison of expression levels of RFP-P2A-GFP and RFP-P2A*-GFP. RFP-P2A-GFP- or RFP-P2A*-GFP-expressing cells were analyzed using flow cytometry, and the GFP fluorescence mean was calculated.

Virus-derived P2A peptide induces protein cleavage during translation^21–23^. Cleaved proteins should be synthesized at the same molecular numbers, because they are translated by the same ribosome. Therefore, by placing GFP at the C-terminal of P2A peptide, the expression level of the target protein placed at the N-terminal of P2A peptide can be indirectly estimated from the GFP expression level. To confirm whether P2A-GFP can be used to estimate the expression level of the N-terminal target protein, we analyzed the expression limit of the red fluorescent protein (RFP) mCherry using the P2A-GFP fusion protein (Fig. 3A). Microscopic observation of transfected cells showed highly correlated RFP and GFP fluorescence (Fig. 3B,C). This high correlation was not due to noncleaved products, because none were observed by western blotting (Fig. 3D,E). These results confirmed that the expression level of the target protein placed at the N-terminal of P2A-GFP can be estimated from the GFP expression level. Fusing proteins with the noncleaving P2A mutant (P2A*) marginally but significantly decreased GFP expression (Fig. 3F; *p* = 0.04, Student’s *t*-test). From the western blotting in Fig. 3E, we could interpret that the GFP fluorescence in RFP-P2A-GFP was all from the cleaved GFP, and that in RFP-P2A*-GFP was from the cleaved and noncleaved GFPs. This indicates that compared to the expression of fusion proteins, cleaved protein expression less perturbs the expression system.

**Figure 4:**
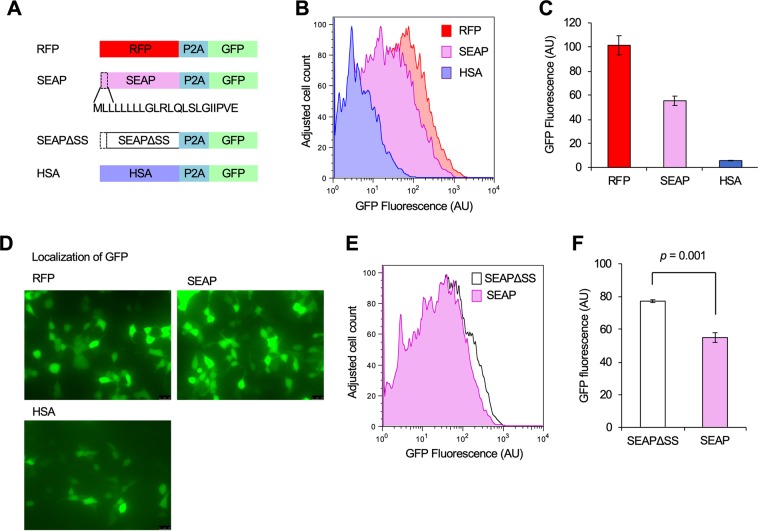
Estimation of expression limits of secretory proteins SEAP and HSA. (**A**) Insert constructions used in the experiment. The SEAP signal sequence is shown as an amino acid sequence. SEAPΔSS represents SEAP without the signal sequence. (**B**,**C**) GFP expression levels of transfected HEK293 cells with the RFP, SEAP and HSA plasmids analyzed using flow cytometry. Histograms of GFP fluorescence (**B**), and the means and SD (error bar) are shown (**C**). (**D**) Microscopic analysis of transfected cells with RFP, SEAP and HSA plasmids. Contrasts of images were emphasized so that the GFP distribution in the cell is visible. (**E**,**F**) GFP expression levels of the cells with Flow cytometry of HEK293 cells with SEAP and SEAPΔSS plasmids analyzed using flow cytometry. Histograms of GFP fluorescence (**E**), and the means and SD (error bar) are shown (**F**). Mean GFP fluorescence of GFP-positive cells obtained by flow cytometry. The flow cytometry histograms were created using the FlowJo software (https://www.flowjo.com/) ver. 8.8.7.

Next, we estimated the expression limits of secreted alkaline phosphatase (SEAP) and human serum albumin (HSA) as nonfluorescent target proteins (Fig. 4A). GFP fluorescence of cells transfected with SEAP and HSA plasmids was 54% and 5% of GFP fluorescence of cells transfected with the RFP plasmid (Fig. 4B,C). Fluorescence decrease seemed not to change in the localization of GFP located at the C-terminal of the secreted proteins, because GFP expressed with these plasmids was observed in the entire cytoplasm but not in the secretion machinery (Fig. 4D). This observation is consistent with the finding that P2A peptide effectively separates the localization of connected proteins^23^. Therefore, C-terminal GFP can be used as a cytoplasmic indicator for the production of N-terminal target proteins localized to other cellular compartments. These results indicated that the expression limits of these proteins are lower compared to RFP, while the expression limit of HSA is only 10% of that of SEAP.

To test whether SEAP’s expression limit is restricted by transport, we added P2A-GFP to SEAPΔSS in which the signal sequence was deleted from SEAP, and estimated the expression limit (SEAPΔSS; Fig. 4A). GFP fluorescence of cells transfected with SEAPΔSS showed a significant increase compared to cells transfected with SEAP (Fig. 4E,F; *p* = 0.001, Student’s *t*-test). This result further confirmed the idea that protein localization decreases the protein expression limit.

### Estimation of expression limits of proteins involved in vesicular transport

**Figure 5:**
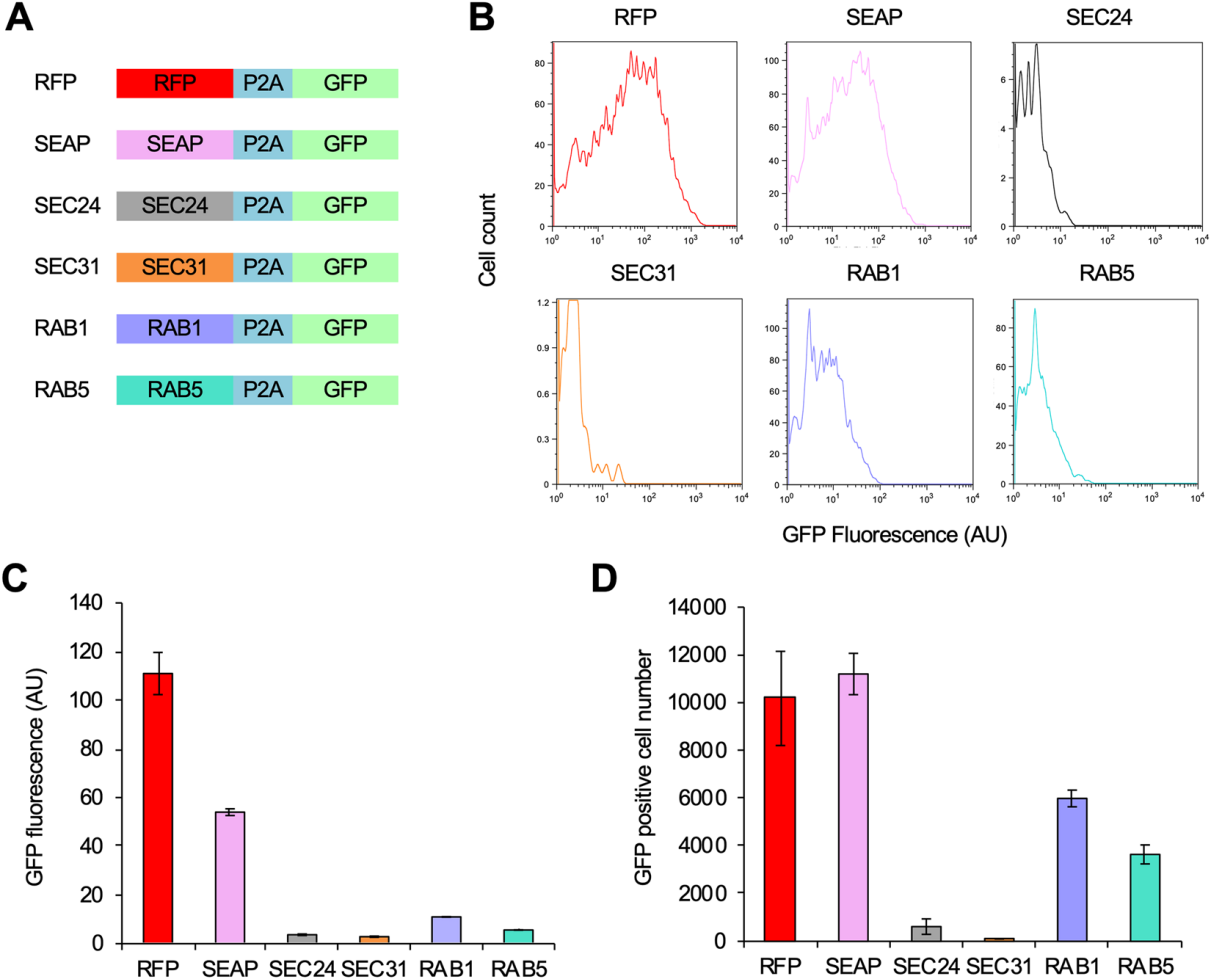
Estimation of expression limits of proteins involved in vesicular transport. (**A**) Insert constructs used in the experiment. (**B**) Flow cytometry of HEK293 cells transfected with each Sec24, Sec31, Rab1, and Rab5 plasmid. (**C**) Mean GFP fluorescence of GFP-positive cells obtained by the flow cytometry in **C**. (**D**) Number of GFP-positive cells in the flow cytometry in (**C**). The flow cytometry histograms were created using the FlowJo software (https://www.flowjo.com/) ver. 8.8.7.

Proteins involved in vesicular transport, such as Sec24, Sec31, Arf1, and Arf2, are toxic when overexpressed in yeast^11^. To test whether overexpression of their human homologs (i.e., Sec24, Sec31, Rab1, and Rab5) is also toxic, we estimated their expression levels using the P2A experimental method (Fig. 5A). GFP fluorescence in the cells transfected with Sec24, Sec31, Rab1, and Rab5 was <1/10 of the GFP fluorescence of cells transfected with RFP and SEAP (Fig. 5B,C). In addition, when transfected with Sec24 and Sec31, the number of surviving cells with GFP fluorescence was <10% of those cells transfected with RFP (Fig. 5D). These results suggested that the expression limits of these proteins involved in vesicular transport are very low, and thus, overexpression of these proteins is toxic, as observed in yeast.

### Concentration of cells with higher plasmid copies using DHFR–MTX antagonism

**Figure 6:**
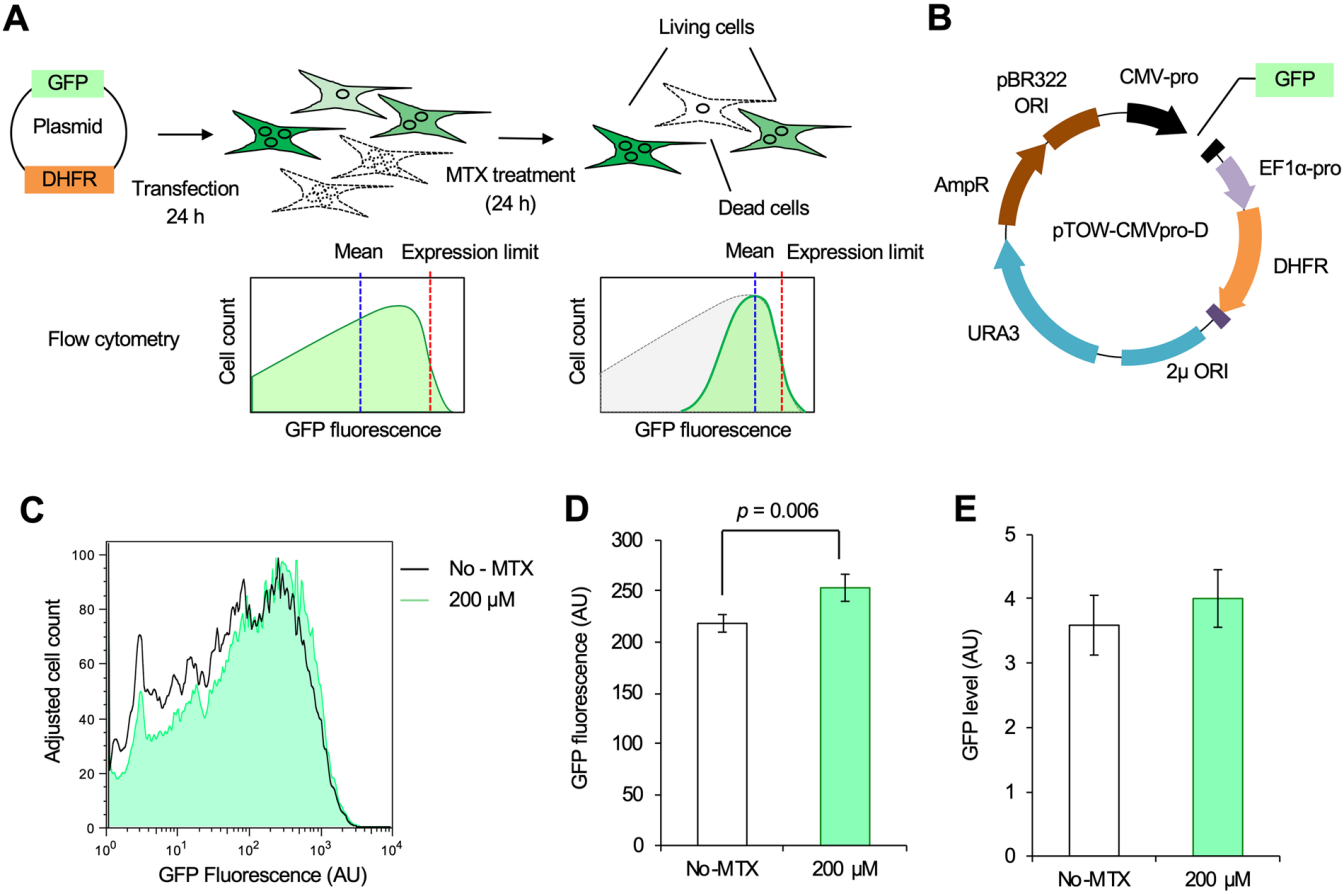
Concentrating cells with higher plasmid copies using DHFR–MTX antagonism. (**A**,**B**) Experimental procedure. Cells are transfected with the plasmid shown in (**B**). After 24 h, MTX is added to the cells and the cells analyzed. Posttreatment, only cells with higher plasmid copies are selected. Virtual histograms in flow cytometry at each step are shown. See the main text for details. (**C**) Flow cytometry of cells transfected with the plasmid GFP-DHFR. The black-lined graph and the filled-green graph indicate cell counts treated with 0 and 200 µM MTX, respectively. (**D**) Mean GFP intensity of GFP-positive cells obtained by flow cytometry in (**C**). (**E**) Mean GFP protein level of cells transfected with GFP-DHFR and treated with indicated MTX. GFP was detected by western blotting. The flow cytometry histograms were created using the FlowJo software (https://www.flowjo.com/) ver. 8.8.7.

So far, we estimated protein expression limits using the excessive efficiency of transfection. However, such transfected cell population contains cells with lower plasmid copy numbers than that required to achieve the protein expression limit. Therefore, the mean GFP expression level analyzed so far should be lower than the actual protein expression limit (Fig. 6A, left).

Therefore, we tried to concentrate those cells in which the target protein’s expression level was closer to the actual protein expression limit by using DHFR–MTX antagonism^24^. The mouse *Dhfr* gene under the control of the EF1-α promoter (EF1α-pro) was inserted into the plasmid pTOW-CMV-pro-D, as shown in Fig. 6B. Transfected cells with lower copy numbers of this plasmid were selectively eliminated by the addition of MTX, and the mean GFP expression level of the surviving cell population came closer to the protein expression limit (Fig. 6A, right). A pilot experiment suggested that treatment with 200 µM MTX eliminates most of the HEK293 cells after 24 h cultivation (data not shown). Therefore, we used 200 µM MTX for subsequent experiments.

As shown in Fig. 6C, among cells transfected with the DHFR-GFP plasmid, 200 µM MTX treatment slightly depleted the portion of cells with lower GFP expression. The mean GFP fluorescence and the GFP protein level by western blotting slightly increased (Fig. 6D,E), and the former increase was statistically significant (*p* = 0.006, Student’s *t*-test). These results suggested that DHFR–MTX antagonism can be potentially used to increase the accuracy of estimation of the protein expression limit.

### Discussion

While protein overexpression causes various cellular defects, the underlying mechanism is unknown. This mechanism is intrinsically associated with the level of overexpressed proteins^3,4^. Therefore, we need to know the protein level that causes cellular defects.

We previously developed the gTOW method to systematically estimate protein expression limits (e.g. expression levels that cause growth defects) in yeasts^8–10^. In the gTOW method, we can genetically estimate a protein’s expression limit using the characteristics of the multicopy plasmids used for gTOW; the intracellular plasmid copy number varies and can be increased using selection bias. Although estimation of the protein expression limit is not precise, because the method uses variance, because of its simplicity, the method can be used to analyze many different samples. In the gTOW method in yeasts, cells harboring higher plasmid copies are concentrated using the selection bias of marker genes. In contrast, concentrating these cells by selection from among cultured cells is not practical because their cell cycles are far longer compared to yeast cells. Therefore, we directly used the variance of high-efficient transfection. In the gTOW method, we used a target gene with its native promoter and terminator so that its expression limit could be estimated as a fold increase from the native level by measuring the plasmid copy number, and therefore, we can obtain the relative overexpression data for each target gene^4^. This is only applicable to organisms whose genome structures are compact: identification of a gene’s promoter region is relatively easy, and genes do not contain large introns. In contrast, in higher eukaryotes, such as mammals, genome structures are much more complex and gene sizes are too large to clone into a plasmid. Therefore, systematic estimation of relative protein overexpression levels for many target genes is not practical.

In this study, we developed a method of estimating and comparing absolute overexpression levels of target proteins. We used self-cleaving P2A peptide so that protein synthesis levels of the marker protein GFP and the target protein were 1:1. We note that their final protein levels would not necessarily be the same when the stabilities of these proteins are not the same (Fig. S1). In fact, while GFP is a stable protein harboring a half-life of many hours^25,26^, about 30% of synthesized proteins in human cells are degraded with a half-life of 1 hour^27^. Therefore, expression limits monitored by the GFP levels in our experiments using P2A peptide should be considered as the relative quantity reflecting the synthesis limits of target proteins rather than the limits of the final protein levels. Technically, the expression levels of target proteins can be directly assessed using western blotting with anti-P2A antibodies. Other possible way to directly assess the target protein level using GFP is to use fusion proteins. However, this might have the negative effects of fusing proteins with a 26 kDa GFP to either N- or C-terminal^28^. The cleaved GFP stayed in the cytoplasm, even if it connected with secreted proteins (Fig. 4D), and fusing GFP by introducing a mutation in P2A peptide negatively affected the expression limit (Fig. 3F).

In our previous work using yeast, we reported two potential mechanisms restricting expression limits of secreted proteins^7^; one is the ER stress where the secreted target protein is misfolded in the ER^29^, and the other is clogging where the target protein blocks the translocation machinery^30^. The normal GFP containing two cysteines aggregates through S-S bond and triggers the ER stress^7^. While moxGFP, a cysteine-free derivative of GFP, does not aggregate in the ER^12^ and thus does not trigger the ER stress, it has the same expression limit as GFP, probably because it clogs the translocation machinery^7^. In this study, we used moxGFP for all experiments to avoid artificial ER stress. The addition of secretion signal to moxGFP dramatically decreased its expression limit to about 10% of that of moxGFP (Fig. 2), probably it triggers clogging in HEK293 cells as well. Interestingly, estimated expression limit of a secreted protein SEAP was about a half of that of a cytoplasmic protein RFP, and removal of the secretion signal from SEAP had a minor effect on its expression limit (Fig. 4). This suggests that SEAP might have a property to avoid the ER stress and clogging.

To overexpress target proteins, we used CMV-pro, which works as the strongest promoter in cultured cells^19,20^. In the experiment to estimate Sec24 and Sec31 expression limits, only a small part of the cells became GFP-positive (i.e., transfection was established) (Fig. 5), suggesting that CMV-pro is too strong to estimate the expression limits of these proteins and that weaker or controllable promoters should be used to estimate very toxic proteins upon their overexpression.

In this study, we used the diversity in transient transfection to survey the expression limits of a target protein (Fig. 1). This method has its own merit that a wide range of expression levels can be surveyed with a single transfection experiment, and thus can be suitable for analyzing various target proteins. Instead, it has a limitation that the measurement of the expression limit is inaccurate. For the precise measurement of the expression limit of a protein, the establishment of the stable cell lines with a controllable gene expression system like Tet-system^31^ or other inducible systems^32^ might be preferable.

We proposed a system of concentrating cells with higher plasmid copies using DHFR–MTX antagonism. However, the system showed only a minor improvement in the measurement (Fig. 6). While the DHFR–MTX system is established to create stable cell lines with high-copy integration^33^, it requires long-term selection and did not seem to work in our transient transfection system.

An interesting finding of this study was that GFP has an expression limit in HEK293 cells. Our estimate was ~5% of total protein (Fig. 2), which was lower than that estimated in yeast (15% of total protein)^7^. However, currently, we cannot conclude whether the difference is due to the difference in cell species or experimental systems. As observed in yeast^7^, adding localization signals to GFP decreased its expression limit (Fig. 2). This finding supported the idea that the burden for localization determines the proteins’ expression limits in cultured cells as well. How does the overexpression of GFP, a gratuitous protein, affect cellular function? The cellular resource for protein production should have a limit, and extreme GFP overexpression overloads the resource. In microorganisms, this situation is called the protein burden/cost and is observed as growth defects^6^. In this study, HEK293 cells overexpressing GFP detached from the bottom of the culture dish, became spherical shaped, and eventually died (Fig. 1H,I). Similar cell death of other cell lines upon overexpression of GFP was also observed previously^34^. The next interesting question is whether there is an intrinsic sensing system of the overloading of protein production. Such a system could work as a programmed suicide system upon viral infection or cancer development, where massive protein production is unrelated to cellular function.

In conclusion, we developed a method of estimating the protein expression limit in cultured cells using only one plasmid system. Because of its simplicity, this method can be applied to various different genes and constructs. It can also be combined to assess the effects of drugs, gene knockdown, and gene overexpression.

## Materials and Methods

### Cell culture and transfection

We used HEK293 cells stocked in our laboratory. HEK293 cells were cultured in Dulbecco’s modified Eagle’s medium (DMEM; Welgene) supplemented with 10% fetal bovine serum (FBS; Gibco). Next, the cells were seeded in 6-well culture plates to be confluent on the following day, with 1.5 mL medium in each well, and then incubated in a CO_2_ incubator overnight. Plasmid DNA and 1 mg/ml PEI “max” (24765–2; Polysciences, Inc.) in a 1:3 weight ratio were mixed with 500 µL of DMEM, and the mixture was incubated for 20 min at room temperature. Finally, the mixture was dropped into each well and incubated in the CO_2_ incubator.

### Plasmids

The plasmids we used in this study are listed in Supplementary Table S1, and their sequences are available upon request. The plasmids were constructed by the homologous recombination activity of yeast cells. CMV-pro, *moxGFP*, and the SV40 polyA signal were obtained from the plasmid moxGFP (plasmid #68070; Addgene). The MTS was from human mitochondrial protein Cox8^35^, and the SS was from *Trypanosoma brucei* EP protein^36,37^.

### Flow cytometry

The transfection efficiency, GFP expression level, and number of dead cells were assessed by flow cytometry using the Cell Lab Quanta flow cytometer (Beckman Coulter). HEK293 cells were centrifuged at 2300 g for 2 min and resuspended in 500 µL of phosphate-buffered saline (PBS, 14249–95; Nacalai tesque). A maximum of 20,000 events (cells) were analyzed per sample. GFP fluorescence was detected using the FL1 channel. The mean GFP fluorescence (AU) of cells was calculated from the GFP fluorescence of GFP-positive cells (FL1 > 10^0.2^), and the mean and SD of AUs from three biological experiments were calculated. We note that the AUs are only comparable within each experiment but not among different experiments, because the sensitivities of the fluorescence detection were altered among experiments.

### Microscopic observation

For cellular images in Fig. 1, cultured cells were directly observed under an Olympus 20-fold objective lens microscope (Olympus). Fluorescence images were observed using the GFP filter cube. Cellar images used in Figs. 2 and 3 were obtained using the DMI6000 B microscope and Leica Application suite X (Leica Microsystems). The mean GFP and RFP fluorescence intensities of each cell obtained by GFP and RFP filter cubes were calculated using a CellProfiler software (ver. 2.2.0, http://cellprofiler.org) pipeline; the pipeline is available upon request.

### MTX treatment for DHFR-expressing HEK293 cells

HEK293 cells were transfected with the plasmid harboring *DHFR* and cultured in a CO_2_ incubator for 24 h. The medium was removed, the cells were washed with 1 mL of PBS, and the medium and MTX (139–13571; FujiFilm Wako Pure Chemical) were added to the wells. The cells were again cultured in the CO_2_ incubator for 48 h. Finally, the cells were analyzed using flow cytometry and western blotting.

### Protein analysis

After removal of the medium, the cells were washed with 1 mL of PBS. The cells were treated with 250 µL of trypsin solution (32778–05; Nacalai tesque), and suspended with 500 µL of PBS. The cells were collected by centrifugation at 2,300 g for 2 min. After removal of the buffer, the cells were suspended with 500 µL of 4 x NuPAGE LDS sample buffer (NP0007; Thermo Fisher Scientific) and incubated at 70 °C for 10 min. The solution was centrifuged at 15, 000 rpm for 5 min. The supernatant then was diluted twice with H_2_O, and used for further analysis as the total protein solution. This treatment completely terminates the fluorescence of GFP (Fig. S2). For visualization of the total protein, the total protein solution was mixed with the Ezlabel FluoroNeo fluorescent dye (WSE-7010; ATTO) at a final concentration 1 x, and incubated at 95 °C for 3 min. The labeled protein was separated using the NuPAGE 4–12% Bis-Tris Gel (NP0322; Thermo Fisher Scientific). The proteins were detected using the LAS-4000 image analyzer (GE Healthcare) in the SYBR-green fluorescence detection mode, and the intensities were measured using the ImageQuant TL software (GE Healthcare). To detect GFP by western blotting, an anti-GFP antibody (11814460001; Roche), a peroxidase-conjugated secondary antibody (414151 F; Nichirei Biosciences), and a chemiluminescence reagent (34095; Thermo Fisher Scientific) were used. A chemiluminescence image was acquired using LAS4000 in the chemiluminescence detection mode, and intensities were measured using the ImageQuant TL software ver. 8.1 (GE Healthcare, https://www.gelifesciences.com/). Calculation of the GFP level over the total protein (%), and the relative expression level of GFP (AU) were performed as shown in Fig. S3. We note that the AUs are only comparable within each experiment but not among different experiments, because we did not use any standard in the western blotting.

### Statistical analysis

Data were analyzed using the FlowJo software (https://www.flowjo.com/) ver. 8.8.7. The mean and SD of the mean GFP intensity of GFP-positive cells were calculated from three independent biological experiments. Two-tailed Student’s *t*-test was used for statistical tests.

## Supplementary information

Supplementary Information.

## Data Availability

The data that support the findings of this study are available from the corresponding author upon reasonable request.
