# Supplementary material for: Development of an experimental method of systematically estimating protein expression limits in HEK293 cells
Source: Sci Rep. 2020 Mar 16;10:4798. doi: 10.1038/s41598-020-61646-3 (PMC7075890; doi:10.1038/s41598-020-61646-3)
Supplement: Supplementary file 1 — Supplementary Information. [file 41598_2020_61646_MOESM1_ESM.pdf]

Supplementary Information (Figure S1-S3, Table S1) for

Development of an experimental method of systematically estimating protein expression limits in HEK293 cells

Yoshihiro Mori[1], Yuki Yoshida[2], Ayano Satoh[3], Hisao Moriya[4,5,*]

1. Graduate School of Natural Science and Technology, Okayama University
2. Sony Computer Science Laboratories
3. Graduate School of Interdisciplinary Science and Engineering in Health Systems, Okayama University
4. Research Core for Interdisciplinary Sciences, Okayama University
5. Graduate School of Environmental and Life Science, Okayama University

* Correspondence to: hisaom@cc.okayama-u.ac.jp

Figure S1

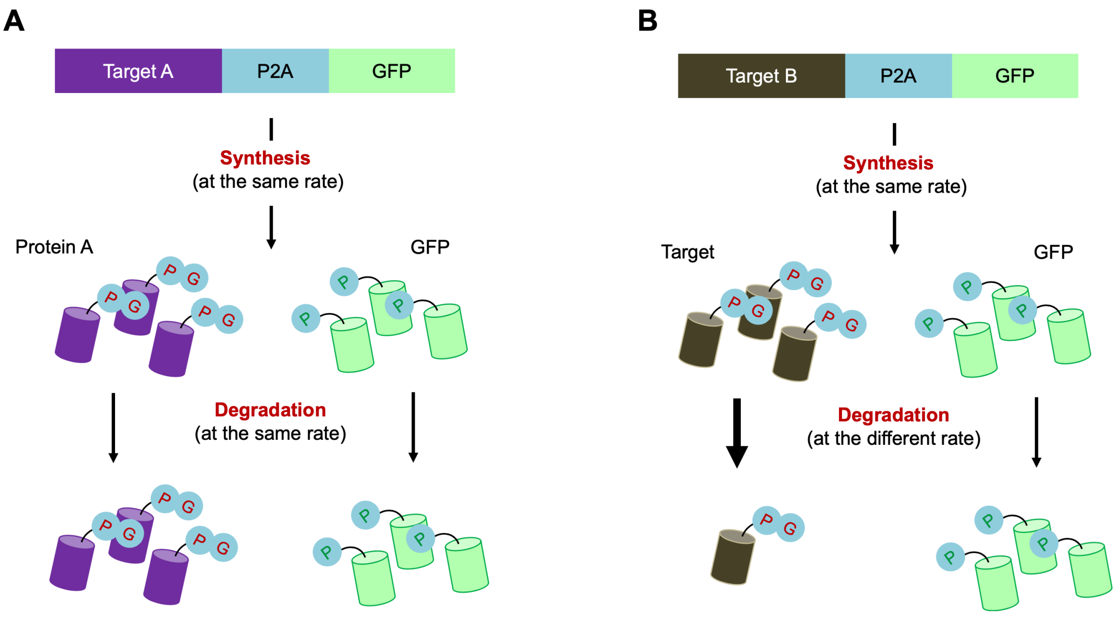

Figure S1. Final levels of P2A-connected proteins are not necessarily the same when half-lives of the proteins are different. (**A**). If protein A is as stable as GFP, the GFP level directly reflects the expression level of protein A. (**B**) If protein B is unstable than GFP, the GFP level does not directly reflect the expression level of protein B.

Figure S2

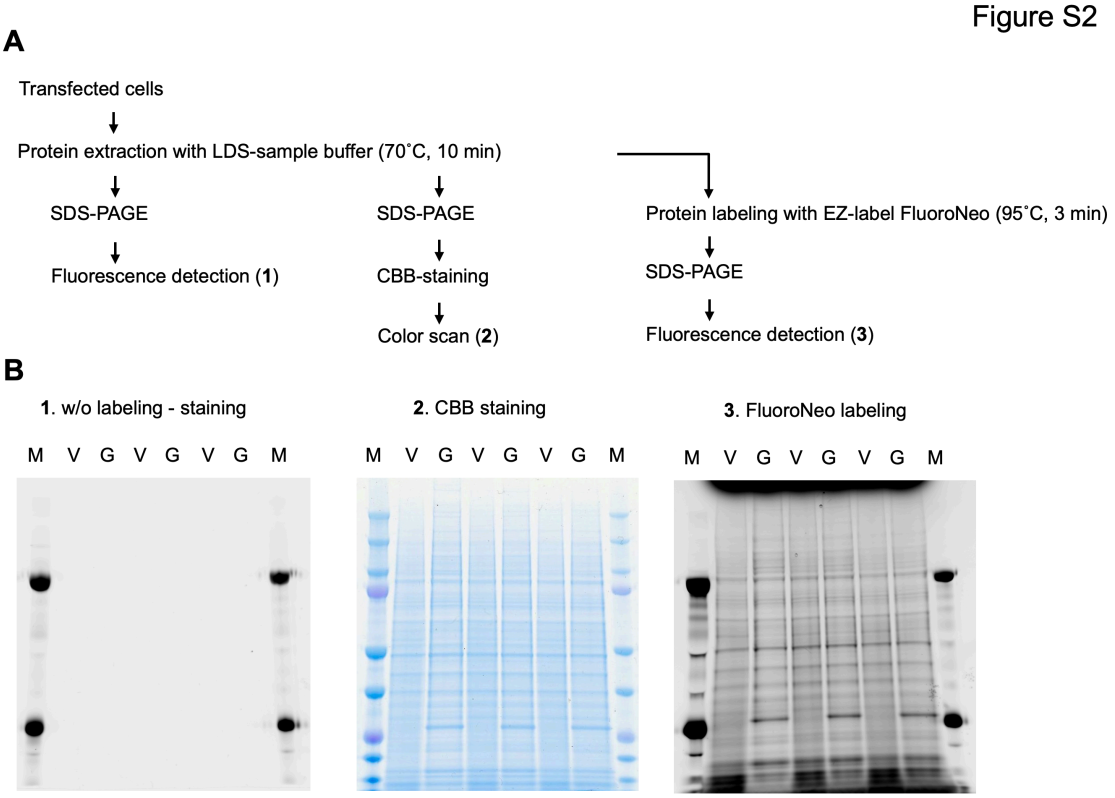

Figure S2. Protein analysis procedure in this study terminate the fluorescence of GFP. (**A**) Scheme of protein analysis. For the measurement of the GFP level in Figure 2 and 6 of this study, we used the scheme 3. (**B**) Gel images analyzed in the scheme in **A**. M, molecular weight marker; V, protein samples of cells transfected with a control vector plasmid pTOW40836; G, protein sample of cells transfected with the GFP-expressing plasmid pCMV-GFP.

Figure S3

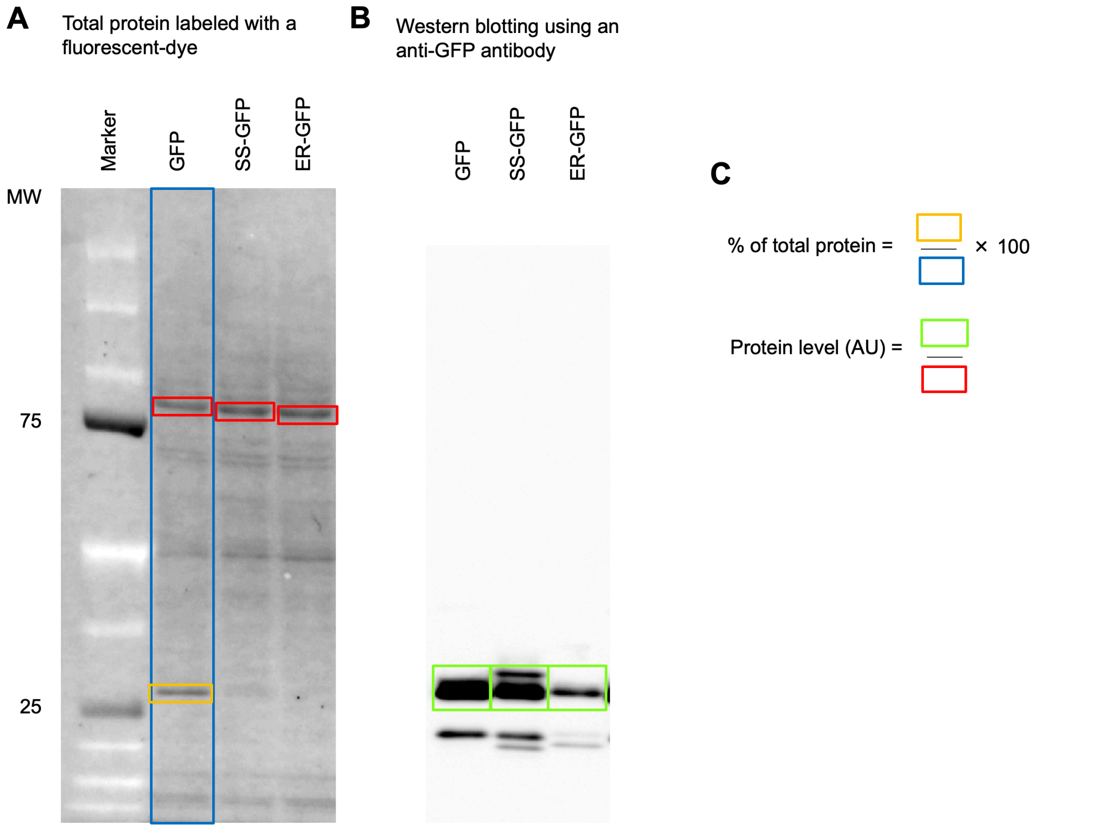

Figure S3. Measurement of the GFP expression level. (A and **B)** Representative gel images used to measure GFP levels. **(C)** Calculation to estimate GFP levels (% of total protein, and AU). The GFP level over the total protein (%) was calculated by dividing the intensity of the GFP band in **A** (orange square) by the intensity of total protein bands in **A** (blue square). The relative GFP levels (AU) were calculated by normalizing the intensities of GFP bands detected by western blotting in **B** (green squares) with the intensities of loading control protein bands in **A** (red squares). The means and SD were calculated from three biological replicates. **B** is the uncropped image of Figure 2F.

Table S1. Plasmid used in this study

Serual No.	Series	Name	Genes	Plasmid backbone	Reference	Note
	Vector					
1		pTOW40836	*2μOri*, *URA3*, *leu2d*, *AmpR*, *ColE1Ori*		Moriya et al., 2012	
	pTOW-CMVpro series					
2		pCMV-GFP	CMV promoter moxGFP	pTOW40836	This study	
3		pCMV-MTS-GFP	CMV promoter MTS-moxGFP	pTOW40836	This study	
4		pCMV-ER-GFP	CMV promoter SS-moxGFP-KDEL	pTOW40836	This study	
	pTOW-CMVpro-P2A series					
5		pCMV-RFP	CMV promoter mCherry-P2A-moxGFP	pTOW40836	This study	
6		pCMV-RFP*	CMV promoter mCherry-P2A mutant-moxGFP	pTOW40836	This study	
7		pCMV-SEAP	CMV promoter SEAP-P2A-moxGFP	pTOW40836	This study	
8		pCMV-HSA	CMV promoter HSA-P2A-GFP	pTOW40836	This study	

9		pCMV-SEAPΔSS	CMV promoter SEAP Δ SS-P2A-moxGFP	pTOW40836	This study	Signal sequence is deleted
10		pCMV-SEC24	CMV promoter SEC24-P2A-moxGFP	pTOW40836	This study	
11		pCMV-SEC31	CMV promoter SEC31-P2A-moxGFP	pTOW40836	This study	
12		pCMV-RAB1	CMV promoter RAB1-P2A-moxGFP	pTOW40836	This study	
13		pCMV-RAB5	CMV promoter RAB5-P2A-moxGFP			
	pTOW-CMVpro-D series					
14		pCMV-GFP-D	CMVpromotter moxGFP, Ef1 α promoterDHFR	pTOW40836	This study	
